# Inhibition of VHL by VH298 Accelerates Pexophagy by Activation of HIF-1α in HeLa Cells

**DOI:** 10.3390/molecules29020482

**Published:** 2024-01-18

**Authors:** Yong Hwan Kim, Na Yeon Park, Doo Sin Jo, Ji-Eun Bae, Joon Bum Kim, Kyuhee Park, Kwiwan Jeong, Pansoo Kim, Eunbyul Yeom, Dong-Hyung Cho

**Affiliations:** 1 School of Life Sciences, BK21 FOUR KNU Creative Bio Research Group, Kyungpook National University, Daegu 41566, Republic of Korea; yoo035913@gmail.com (Y.H.K.);; 2 ORGASIS Corp., Suwon 16229, Republic of Korea; 3 KNU LAMP Research Center, KNU Institute of Basic Sciences, College of Natural Sciences, Kyungpook National University, Daegu 41566, Republic of Korea; 4 Bio Industry Department, Gyeonggido Business & Science Accelerator, Suwon 16229, Republic of Korea; 5 Organelle Institute, Kyungpook National University, Daegu 41566, Republic of Korea

**Keywords:** VH298, VHL E3 ligase, HIF-α, autophagy, pexophagy

## Abstract

Autophagy is a pivotal biological process responsible for maintaining the homeostasis of intracellular organelles. Yet the molecular intricacies of peroxisomal autophagy (pexophagy) remain largely elusive. From a ubiquitin-related chemical library for screening, we identified several inhibitors of the Von Hippel–Lindau (VHL) E3 ligase, including VH298, thereby serving as potent inducers of pexophagy. In this study, we observed that VH298 stimulates peroxisomal degradation by ATG5 dependently and escalates the ubiquitination of the peroxisomal membrane protein ABCD3. Interestingly, the ablation of NBR1 is similar to the curtailed peroxisomal degradation in VH298-treated cells. We also found that the pexophagy induced by VH298 is impeded upon the suppression of gene expression by the translation inhibitor cycloheximide. Beyond VHL inhibition, we discovered that roxadustat, a direct inhibitor of HIF-α prolyl hydroxylase, is also a potent inducer of pexophagy. Furthermore, we found that VH298-mediated pexophagy is blocked by silencing *HIF-1α.* In conclusion, our findings suggest that VH298 promotes pexophagy by modulating VHL-mediated HIF-α transcriptional activity.

## 1. Introduction

Peroxisomes, working synergistically with mitochondria, are metabolic powerhouses responsible for vital biological processes, including the β-oxidation of fatty acids, lipid biosynthesis, and regulation of reactive oxygen species (ROS) metabolism [1,2,3]. They further serve as integral platforms for immune-response signaling [4]. Therefore, the malfunction of peroxisomes has been implicated in an array of human diseases, including metabolic disorders, cancers, and neurodegenerative diseases [5,6]. Peroxisomes, which are dynamic cellular organelles, exhibit extensive variability in their content, shape, size, and number across various cell types [1]. To maintain the optimal function and number of peroxisomes, their quality and quantity are tightly regulated by peroxisome biogenesis and peroxisome degradation [1,6,7]. Mutations in various peroxisomal biogenesis factors, such as peroxins, lead to a spectrum of peroxisomal biogenesis disorders, including Zellweger syndrome [8,9,10]. Various peroxins are synthesized in the cytosol and are subsequently targeted to the peroxisome through the recognition of peroxisomal targeting signals (PTS1, PTS2, or mPTS) [1]. Most peroxins feature a conserved tripeptide sequence (PTS1) at their C-terminus. Additionally, a select group of peroxisomal matrix proteins are identified by the presence of a PTS2 motif located at the N-terminus. Moreover, some peroxisomal membrane proteins (PMPs) are directly localized to the peroxisome through the membrane PTS (mPTS) [1,7]. Current research on these proteins and their associated pathways deepens our understanding of peroxisome function and reveals potential targets for therapeutic interventions [11].

Autophagy is an essential cellular recycling pathway that degrades superfluous or defective cellular components including organelles [12,13,14]. The optimal acidic pH within the lysosomal lumen enhances the activity of lysosomal hydrolytic enzymes, enabling the degradation of cellular components [12]. Autophagy is controlled by various autophagy-related (ATG) proteins, among which ATG5 and ATG7 play crucial roles [12,13]. The microtubule-associated protein 1A/1B-light chain-3-I (LC3-I) undergoes conversion into LC3-II, a form that is recruited to autophagosomal membranes [13,15]. This ensuing fusion of autophagosomes with lysosomes facilitates the clearance of cellular components [13]. Autophagic flux can be effectively monitored by using the fluorescence protein system (monomeric red fluorescent protein (mRFP)-enhanced green fluorescent protein (EGFP) tandem system) [12,15]. This system is based on the unique spectral characteristics and pH sensitivity of EGFPs and mRFPs. Pexophagy is a highly selective autophagic process that orchestrates the targeted degradation of peroxisomes [16,17,18]. This process becomes increasingly relevant under stress conditions, such as starvation and hypoxia, wherein it facilitates the systematic removal of superfluous or dysfunctional peroxisomes, ensuring cellular homeostasis [16,19,20]. Our understanding of the molecular mechanisms underlying pexophagy has evolved with recent studies, highlighting the indispensable role of the ubiquitination of peroxisomal membrane proteins. In particular, ataxia telangiectasia mutated (ATM) kinase has emerged as a central regulator in this process [21]. When the cell is exposed to high ROS levels, ATM kinase is activated, increasing the phosphorylation of the peroxisomal protein PEX5, which further facilitates the ubiquitination of PEX5 for pexophagy [21,22]. ABCD3, also known as peroxisomal membrane protein 70, plays a pivotal role in peroxisome function and is involved in pexophagy [16,23]. Under cellular stress conditions, such as oxidative stress or nutrient deprivation, ABCD3 is ubiquitinated, and the ubiquitinated peroxisomal proteins recruit autophagy adaptors such as p62/SQSTM1 and NBR1 [16,24,25]. Moreover, in conditions of amino acid deficiency, the mitochondrial E3 ubiquitin ligase MARCH5 relocated to the peroxisome, where it ubiquitinates ABCD3 [18]. The recognition and interaction of ubiquitinated proteins and autophagy adaptors sets the stage for autophagosome recruitment [26,27]. Through this complicated pathway, the cell ensures the appropriate management of its peroxisomal population, particularly under stress conditions.

Although various pexophagy inducers have been identified by us and other research groups, the molecular mechanisms underlying pexophagy in mammals remain largely elusive. In this study, we newly identified several inhibitors of VHL E3 ligase, including VH298, as inducers of pexophagy. We revealed that the inhibition of VHL significantly promotes the loss of peroxisomes. Interestingly, this effect is entirely reversible by the depletion of *ATG5* and *NBR1*. Moreover, we demonstrated that VH298 promotes pexophagy by modulating VHL-mediated HIF-α transcriptional activity.

## 2. Results

### 2.1. Inhibition of VHL Promotes Pexophagy

**Figure 1:**
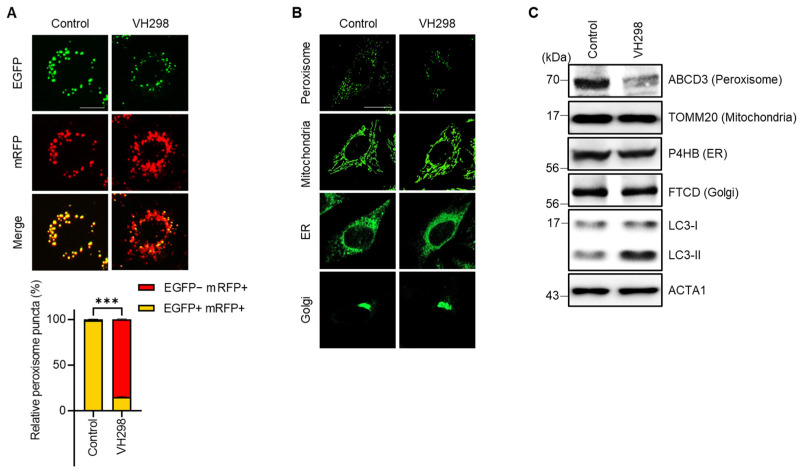
VH298 promotes peroxisome reduction. (**A**) RPE/mRFP–EGFP–SKL cells were treated with VH298 (50 µM) for 48 h and then imaged under a fluorescence microscope. The percentage of puncta exhibiting EGFP(+)- and mRFP(+)-labeled autophagosomes or EGFP(−)- and mRFP(+)-labeled autolysosomes per cell was determined. The scale bar represents 20 µm (data are presented as mean ± standard error of the mean; *n* = 100, and *** *p* < 0.001). (**B**) HeLa cells stably expressing pmTurquiose2-Peroxi, pmTurquiose2-Mito, pmTurquiose2-ER, or pmTurquiose2-Golgi were imaged via confocal microscopy. The scale bar represents 20 µm. (**C**) HeLa cells treated with VH298 (50 µM) for 48 h were subjected to Western blotting by using the indicated antibodies against the protein markers of subcellular organelles (ABCD3 for peroxisomes, TOMM20 for mitochondria, P4HB for the ER, and FTCD for the Golgi apparatus).

Pexophagy serves as a vital mechanism for the maintenance of peroxisome quality. In our previous research, we attempted to reveal new pexophagy regulators through the screening of a chemical library composed of ubiquitination-related small molecules [28]. We utilized hTERT RPE-1 cells consistently expressing the mRFP–EGFP–SKL protein (RPE/mRFP–EGFP–SKL) and identified a few VHL inhibitors, including VH298. Although a previous study reported that the depletion of the tumor suppressor VHL in hepatocytes decreases the number of peroxisomes [19], the effect of VH298 on pexophagy remains unelucidated. Therefore, in this study, we explored the potential effects of VHL on pexophagy. To corroborate the findings from our initial screening, we treated RPE/mRFP–EGFP–SKL cells with VH298 and monitored pexophagy by using the mRFP–EGFP tandem system. Figure 1A indicates the presence of yellow puncta (representing peroxisomes), which were visible due to the confluence of (EGFP[+]/mRFP[+]) double-positive fluorescence. In contrast, cells treated with VH298 showed a significant increase in the number of red-only puncta, which showed a reduced green signal (EGFP[−]/mRFP[+]) (Figure 1A). To further examine selective autophagy at the organelle level, we tracked other cellular structures, including the ER, Golgi apparatus, and mitochondria, in VH298-treated cells. HeLa cells exhibiting the stable expression of pmTurquiose2-Mito, pmTurquiose2-ER, pmTurquiose2-Golgi, or pmTurquiose2-Peroxi were subjected to VH298 treatment, and the intracellular organelles were subsequently visualized. Among the examined organelles, only the population of peroxisomes demonstrated a marked reduction, whereas the populations of the mitochondria, ER, and Golgi apparatus remained unchanged after VH298 exposure (Figure 1B,C). This suggests that VH298 selectively fosters peroxisomal degradation.

### 2.2. Depletion of ATG5 or NBR1 Impedes VH298-Induced Peroxisomal Autophagy in HeLa Cells

**Figure 2:**
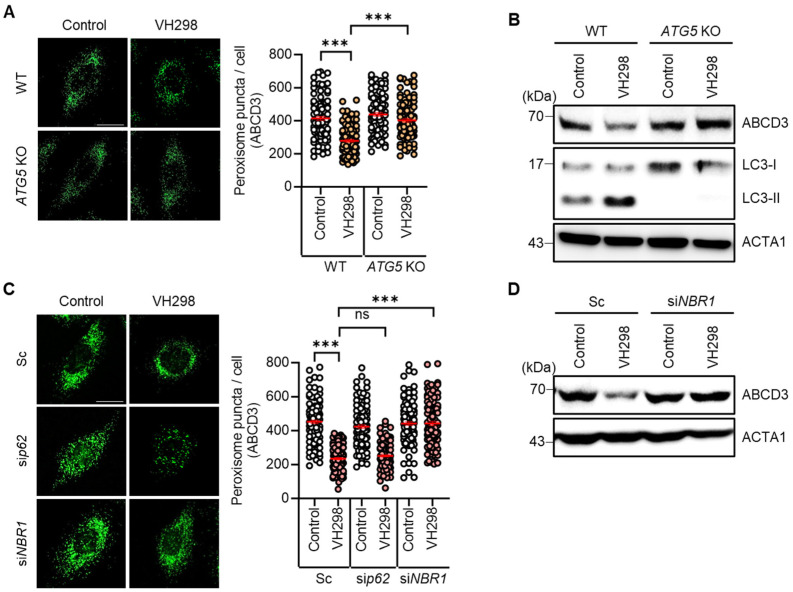
*ATG5* or *NBR1* depletion prevents VH298-induced peroxisome loss. (**A**) Wild-type and *ATG5*-KO HeLa cells were treated with VH298 (50 µM) for 48 h and then stained with anti-ABCD3 antibody. The number of peroxisomes per cell was estimated by analyzing approximately 100 cells. (**B**) After treatment with VH298 (50 µM) for 48 h, wild-type and *ATG5*-KO HeLa cells were subjected to Western blotting by using the indicated antibodies. (**C**) HeLa cells, which were transiently transfected with scrambled siRNA (Sc), siRNA targeting *p62* (si*p62*), or *NBR1* (si*NBR1*), were treated with or without VH298 (50 µM) for 48 h. Subsequently, cells were stained with anti-ABCD3 antibody, and the number of peroxisomes per cell was counted in approximately 100 cells. The scale bar indicates 20 µm (data are presented as mean ± standard error of the mean; *** *p* < 0.001, ns: not significant). (**D**) HeLa cells were treated with VH298 (50 µM) in the presence or absence of validated siRNA against *NBR1* (si*NBR1*) and then harvested for analysis by Western blotting with the indicated antibodies.

We observed a reduction in the number of peroxisomes in VH298-treated cells. Considering that pexophagy refers to the selective autophagic degradation of peroxisomes, we further investigated the influence of VH298 on autophagy in HeLa cells. Both wild-type and *ATG5*-KO HeLa cells were treated with VH298, and the resultant peroxisome loss was assessed. Notably, VH298 significantly induced the loss of peroxisomes in wild-type HeLa cells; however, this effect was entirely blocked in *ATG5*-KO cells (Figure 2A,B). Furthermore, we analyzed the levels of the peroxisomal membrane protein ABCD3 and revealed that *ATG5*-KO effectively prevented the reduction in the levels of ABCD3 in VH298-treated cells (Figure 2A,B). Thus, our findings suggest that VH298 stimulates pexophagy via the ATG5-dependent canonical autophagic pathway. During selective autophagy, the identification of ubiquitinated cargo relies on specific autophagic adaptors, which accelerate the recruitment of autophagosomes for engulfment. It was previously reported that p62/SQSTM1 and NBR1 predominantly act as adaptors for pexophagy [16,24]. NBR1 tends to be more specialized in pexophagy, whereas p62 plays a more multifaceted role in the cell. Accordingly, we further determined the adaptor proteins that play a role in VH298-treated cells, as shown in Figure 2C,D. In agreement with the results of the *ATG5*-KO cells, the depletion of *NBR1*, but not *p62*, significantly inhibited the loss of peroxisomes in VH298-treated cells (Figure 2C,D).

**Figure 3:**
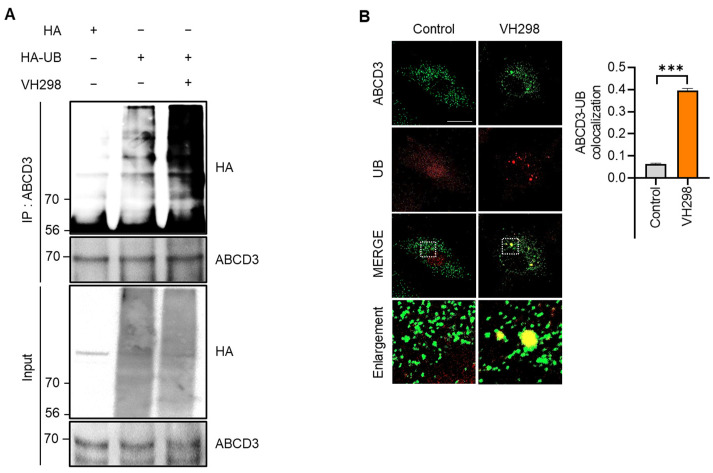
VH298 promotes ABCD3 ubiquitination-mediated pexophagy. (**A**) HeLa cells overexpressing HA and HA-UB were treated with VH298 (50 µM) for 48 h. Following treatment, the cells were harvested and subjected to immunoprecipitation by using anti-ABCD3 antibodies conjugated to agarose beads. The samples were analyzed via Western blotting by using the indicated antibodies. (**B**) HeLa cells were treated with VH298 (50 µM) for 48 h and subsequently stained with anti-ABCD3 (green) and anti-UB (red) antibodies. Pearson’s correlation coefficient was calculated to analyze the colocalization between ABCD3 and UB. The bars in the graph represent the results of colocalization analysis by using the Coloc 2 plugin in Fiji, utilizing Pearson’s correlation coefficient. The mean Pearson’s correlation coefficient values for the level of colocalization between ABCD3 and UB are indicated in the graph. The scale bar indicates 20 µm (data are presented as mean ± standard error of the mean; *n* = 100 and *** *p* < 0.001).

In selective autophagy, target cargo is recognized via the ubiquitination of membrane proteins [29]. It was previously reported that ABCD3 is a ubiquitinated peroxisomal target protein in pexophagy [16,23]. Consistently, we observed that the ubiquitination assay revealed the accelerated ubiquitination of ABCD3 in response to VH298 treatment in HeLa cells (Figure 3A). Moreover, the colocalization of ubiquitin (UB) and ABCD3 was significantly enhanced in VH298-treated cells (Figure 3B). Taken together, these findings suggest that VH298 induces pexophagy through ABCD3- and NBR1-dependent canonical autophagy pathways.

### 2.3. VH298 Promotes Pexophagy by Enhancing the Transcriptional Activation of HIF-1α in RPE Cells

**Figure 4:**
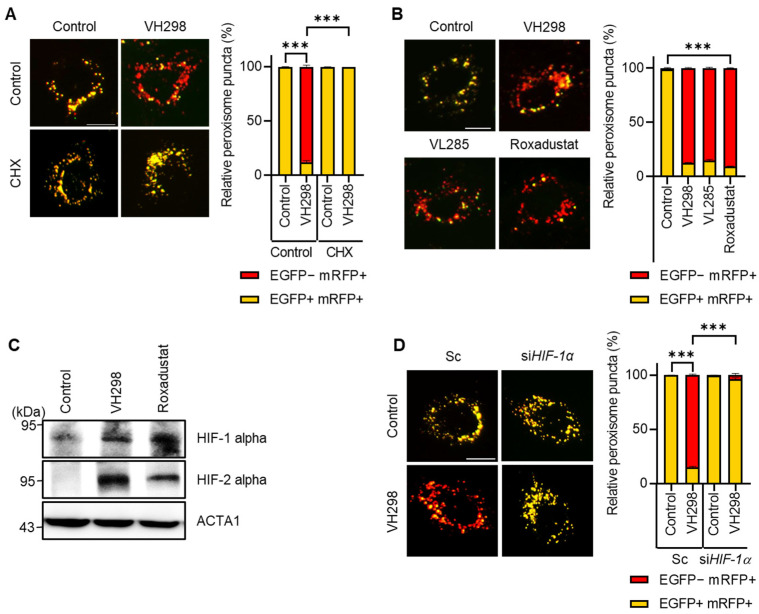
VH298 induces pexophagy via HIF-1α activation. (**A**) RPE/mRFP–EGFP–SKL cells pretreated with VH298 (50 µM) for 42 h were further incubated with or without cycloheximide (10 µg/mL) for 6 h. The cells were then imaged by using a fluorescence microscope. The percentage of puncta exhibiting EGFP(+)- and mRFP(+)-labeled autophagosomes or EGFP(−)- and mRFP(+)-labeled autolysosomes per cell was determined. The scale bar indicates 20 µm (data are presented as mean ± standard error of the mean; *n* = 100 and *** *p* < 0.001). (**B**) RPE/mRFP–EGFP–SKL cells were treated with VH298 (50 µM), VL285 (50 µM), and roxadustat (50 µM) for 48 h and then imaged by using a fluorescence microscope. Subsequently, the fluorescence intensities of EGFP and mRFP were captured and quantified. The scale bar indicates 20 µm (data are presented as mean ± standard error of the mean; *n* = 100 and *** *p* < 0.001). (**C**) RPE cells were treated with VH298 (50 µM) and roxadustat (50 µM) for 48 h, after which they were analyzed via Western blotting by using the indicated antibodies. (**D**) RPE/mRFP-EGFP-SKL cells were transiently transfected with scrambled siRNA (Sc), siRNA targeting *HIF-1α* for 48 h, and then treated with or without VH298 (50 µM) for 48 h. The cells were then imaged by using a fluorescence microscope. The percentage of puncta exhibiting EGFP(+)- and mRFP(+)-labeled autophagosomes or EGFP(−)- and mRFP(+)-labeled autolysosomes per cell was determined. The scale bar indicates 20 µm (data are presented as mean ± standard error of the mean; *n* = 100 and *** *p* < 0.001).

VH298 stabilizes HIF-α, an oxygen-dependent transcriptional activator. We hypothesized that VH298 induces pexophagy by increasing target-gene expression. To test this hypothesis, we treated HeLa cells with VH298 in the presence or absence of cycloheximide, a well-known translation inhibitor. As shown in Figure 4A, we revealed that cotreatment with cycloheximide entirely blocked pexophagy in the VH298-treated cells, suggesting that VH298 promotes pexophagy by modulating gene expression. Next, we directly investigated the effect of HIF-1α activation by treating cells with roxadustat, a HIF stabilizer, and inhibiting the expression of HIF-1α prolyl hydroxylase (PHD) that hydroxylates HIF-1α for degradation. Consistent with the findings of VHL inhibitors, such as VH298 and VH285, the roxadustat treatment strongly promoted pexophagy in RPE cells (Figure 4B,C). Additionally, we noted that the depletion of *HIF-1α* significantly inhibited the reduction in pexophagy in VH298-treated cells (Figure 4D). This observation suggests that silencing *HIF-1α* attenuated the VH298-promoted pexophagy. Taken together, our results demonstrate that gene expression regulated by HIF-1α is crucial for controlling VH298-induced pexophagy.

## 3. Discussion

Peroxisomes play a role in producing signaling molecules that influence tumorigenesis, and pexophagy may act as a regulatory mechanism, either facilitating or hindering cancer progression depending on the situation. The VHL protein plays a central role in the regulation of HIFs, and its inhibition has emerged as a promising therapeutic approach in various pathological states, notably in specific cancers [30,31,32]. In humans, the HIF family includes HIF-1, HIF-2, and HIF-3, each playing distinct roles in hypoxia. Walter et al. demonstrated that autophagy is induced in *VHL*-KO mice in a HIF-2α-dependent manner [19]. They reported that HIF-2α signaling inhibits peroxisome metabolism and amplifies peroxisome degradation, suggesting that VHL plays a regulatory role in peroxisomal abundance and activity. Intriguingly, the altered lipid composition, such as increased levels of very-long-chain fatty acids and reduced levels of docosahexaenoic acid and arachidonic acid, is suggestive of peroxisomal disorders [19]. According to this notion, we revealed that VH298 strongly induces pexophagy without affecting other organelles (Figure 1). VH298-induced pexophagy is linked to HIF transcriptional activity, a key response to hypoxia (Figure 4), elucidating a vital molecular association between VHL-mediated HIF transcriptional activation and pexophagy initiation. HIFs, as transcription factors, activate target-gene expression in response to low oxygen levels by regulating the genes involved in energy metabolism, angiogenesis, and cell survival [33,34]. By activating specific targets such as erythropoietin and vascular endothelial growth factor, HIF enables the cell to adapt to hypoxic conditions, which is vital in physiological processes and pathologies, including cancer [33,35]. In this study, we revealed that the inhibition of gene expression by cycloheximide entirely blocked pexophagy in VH298-treated cells, indicating that VH298-induced pexophagy is controlled by the HIF regulation of target-gene expression (Figure 4). As HIFs target the numerous genes involved in energy metabolism, angiogenesis, and cell survival [33,34], further research on target identification is warranted. Additionally, a previous study reported that dimethyloxaloylglycine (DMOG), an inhibitor that stabilizes hypoxia-inducible factors, promotes pexophagy in a HIF-2α-dependent manner [36]. The loss of HIF-2α inhibited the clearance of peroxisomes and prevented the formation of autophagosomes in DMOG-treated cells. Consistent with these findings, we observed that the activation of HIF by roxadustat significantly induced pexophagy in HeLa cells (Figure 4). Roxadustat, a stabilizer of HIF that inhibits PHD, has been approved as a first-in-class drug for renal anemia treatment [30]. However, roxadustat primarily increases HIF-1α rather than HIF-2α [37,38]. Consistently, treatment with roxadustat significantly increased the level of HIF-1α in aortic tissues, and roxadustat enhanced hepatic HIF-1α stabilization by directly targeting retinal tissue [37,38].

Various studies have shown that HIF-1α upregulates the expression of the mitophagy receptor protein BNIP3L/NIX to promote mitochondrial clearance under hypoxic conditions in several cancer cell lines [39,40,41,42]. BNIP3L-dependent mitophagy leads to a metabolic shift toward glycolysis [40]. Notably, recent reports have suggested that BNIP3L regulates pexophagy as well as mitophagy [43,44]. Wilhelm et al. showed that BNIP3L translocates to or accumulates in peroxisomes upon DFP treatment. As a receptor protein, BNIP3L directly binds to LC3, recruiting autophagosomes during both pexophagy and mitophagy. Nevertheless, selective autophagy, including pexophagy, indicated that the ubiquitination of organelle membrane proteins is the primary pathway for specific cargo. In a previous study, it was reported that under amino acid deficiency, the ubiquitination of ABCD3 by the PEX2 E3 ubiquitin ligase complex PEX2-PEX10-PEX12 triggers an NBR1-mediated pexophagy mechanism [16]. Furthermore, the mitochondrial E3 ligase MARCH5 relocates to the peroxisome to ubiquitinate ABCD3 [18]. In line with these findings, we revealed that UB was translocated to peroxisomes and that the ubiquitination of the peroxisome membrane protein ABCD3 is significantly increased following VH298 treatment (Figure 3). This finding also suggests that VH298-mediated pexophagy utilizes a UB-dependent pathway. However, our study did not address the ubiquitination of other peroxisomal proteins; we focused solely on the ubiquitination of ABCD3, which is the most well-characterized target protein in VHL inhibition by VH298. However, it has been demonstrated that other peroxisomal proteins, like PEX5, are ubiquitinated through an alternative pexophagy pathway [21]. Consequently, it is possible that additional peroxisomal membrane proteins may be involved, warranting further investigation to better understand the molecular mechanism. Moreover, recent advancements in autophagy research have shown that both p62 and NBR1 are involved in peroxisome degradation. The depletion of *p62* inhibits DMOG-induced pexophagy [36]. In contrast to DMOG, VH298, but not p62, promotes pexophagy in an NBR1-dependent manner. We revealed that the loss of peroxisomes was reversed by *NBR1* depletion but not by *p62* knockdown (Figure 2). Thus, further research is warranted to determine the importance of NBR1 recruitment to peroxisomes to facilitate pexophagy.

In hypoxic cancer environments, changes in ROS levels may affect cell signaling, leading to a more aggressive phenotype [45]. The regulation of HIF-mediated pexophagy may act as a mechanism to fine-tune ROS levels, adapt to hypoxia, and further affect cancer progression [21,46]. Additionally, alterations in pexophagy have been linked to numerous pathological conditions, including neurodegenerative and specific metabolic disorders, as well as cancer [4,25,47]. Therefore, a more profound understanding of how HIFs regulate pexophagy might lead to the development of innovative therapeutic strategies for such disorders.

## 4. Materials and Methods

### 4.1. Reagents, siRNAs and Plasmids

A ubiquitination compound library (L8600) for drug screening was obtained from TargetMol (Boston, MA, USA). VH298 (SML1896) and cycloheximide (01810) were purchased from Sigma-Aldrich (St. Louis, MO, USA). Roxadustat (15294) was obtained from Cayman Chemical Co. (Ann Arbor, MI, USA). VL285 (S0095) was obtained from Selleck Chemicals (Houston, TX, USA). Short interfering RNA (siRNA) targeting *NBR1* (5′-GAAGAGGUAUCCAUCAACAUU-3′), *p62/SQSTM1* (5′-GCAUUGAAGUUGAUAUCGAUUU-3′), *HIF-1α* (5’-CAAUCAAGAAGUUGCAUUA-3’), and scrambled siRNA (5′-CCUACGCCACCAAUUUCGU-3′) were synthesized by Genolution (Seoul, Republic of Korea). The subcellular localization vectors, pmTurquoise2-ER, pmTurquoise2-Golgi, pmTurquiose2-Mito, and pmTurquoise2-Peroxi, in which a cyan fluorescence protein (Turquoise) was fused with an ER, Golgi, mitochondria, or peroxisome-targeting sequence, respectively, were obtained from Addgene (36204, 36205, 36208, and 36203; deposited by Dorus Gadella). pcDNA3.1-HA (HA) was obtained from Addgene (128034; deposited by Oskar Laur). HA-tagged ubiquitin WT (HA-UB) was obtained from Addgene (17608; deposited by Ted Dawson).

### 4.2. Cell Culture and Establishment of Stable Cell Lines

hTERT RPE-1 cells stably expressing monomeric red fluorescent protein (mRFP)-enhanced green fluorescent protein (EGFP)-serine-lysine-leucine (SKL) (RPE/mRFP-EGFP-SKL) were kindly provided by Dr. R. Park (GIST, Gwangju, Republic of Korea) [28]. HeLa cells were obtained from the American Type Culture Collection (Manassas, VA, USA). *ATG5*-knockout (KO) HeLa cells generated by using the CRISPR/Cas9 system were kindly provided by Dr. T. Kanki (Niigata University, Niigata, Japan) [29]. All cells were cultured at 37 °C in an incubator under 5% CO_2_ and maintained in DMEM supplemented with 10% FBS (SH30243.01 and SH30084.03; Hyclone, Logan, UT, USA) and 1% penicillin–streptomycin (15140122; Invitrogen, Waltham, MA, USA). To generate stable cell lines, HeLa cells were transfected with pmTurquoise2-Peroxi (HeLa/Peroxi), pmTurquiose2-Mito (HeLa/Mito), pmTurquoise2-Golgi (HeLa/Golgi), and pmTurquoise2-ER (HeLa/ER) by using Lipofectamine 2000 (11668019; Invitrogen), in accordance with the manufacturer’s protocol. Stable transfectants were established via culture in a selection medium containing 1 mg/mL G418 (10131035; Invitrogen) for 7 days. After seeding the individual cells, stable clones were identified under a fluorescence microscope (IX71; Olympus, Tokyo, Japan).

### 4.3. Confocal Microscopy

Stable cell lines expressing fluorescent proteins were cultured on a glass cover slip, treated with VH298 at different times, washed with PBS, and fixed with 4% paraformaldehyde for 20 min. Around 100 fluorescent images of the subcellular localization and morphology of peroxisomes were obtained by using a confocal laser scanning microscope (LSM 800; Carl Zeiss, Oberkochen, Germany).

### 4.4. Determination of Pexophagic Cells

To quantify the cells undergoing pexophagy, HeLa/Peroxi and HeLa cells were seeded on a glass cover slip and treated with VH298 at different times. The cells were then washed with PBS (LB 001-02; WELGENE, Daegu, Republic of Korea), fixed with 4% paraformaldehyde (P2031; Biosesang, Yongin-si, Republic of Korea) for 20 min, and stained with the indicated dyes or antibodies. Punctate peroxisome structures were visually identified by using confocal microscopy (LSM800; Carl Zeiss). The number of peroxisomal structural (ABCD3) puncta was quantified by using ImageJ Fiji (NIH, Bethesda, MD, USA), and the experiments were performed at least three times for each condition, as indicated in each figure. The number of peroxisomes was calculated by dividing the number of peroxisomal structures by the volume of the cell. The same method was used to quantify the ABCD3 puncta.

### 4.5. Quantification of ABCD3-UB Colocalization

To assess the colocalization of ABCD3 and UB, HeLa cells were cultured on a glass cover slip and treated with VH298 for 48 h. The cells were then washed with PBS (LB 001–02; WELGENE, Republic of Korea), fixed with 4% paraformaldehyde (P2031; Biosesang, Korea) for 20 min, and stained with the antibodies (anti-ABCD3 (ab3421; Abcam, Cambridge, UK) and anti-Ubiquitin (sc-8017; Santa Cruz Biotechnology, Dallas, TX, USA)). Fluorescent images of the morphology of peroxisomes and UB were obtained by using a confocal laser scanning microscope (LSM 800; Carl Zeiss). For the analysis of the colocalization between ABCD3 and UB, the Coloc2 plugin in the free software ImageJ Fiji 64-bit (NIH) was utilized. The calculation of double-fluorescence correlation coefficients was performed through the application of Pearson’s correlation coefficient in the Coloc2 plugin as a measure of the association between the two signals (ABCD3-UB). The mean Pearson’s correlation coefficient (R) values for the level of colocalization between ABCD3 and UB are represented graphically.

### 4.6. Western Blotting

Cell lysates were prepared by using a 2× Laemmli sample buffer (1610737; Bio-Rad, Hercules, CA, USA). The total protein quantity was measured by using the Bradford dye (5000001; Bio-Rad) in accordance with the manufacturer’s instructions. The samples were then separated via sodium dodecyl sulfate (SDS)–polyacrylamide gel electrophoresis and transferred onto a PVDF membrane (1620177; Bio-Rad). After blocking with 4% skim milk (90002-594; BD Bioscience, Dubai, UAE) prepared in TBST (Tris base (T9200; GenDEPOT, Baker, TX, USA), NaCl (G0610; GenDEPOT), and Tween^®^ 20 (P7949; Sigma-Aldrich)), the membrane was incubated with the indicated primary antibodies. Anti-ABCD3 (ab3421), anti-P4HB (ab2792), and anti-FTCD (ab27043) antibodies were purchased from Abcam (Cambridge, UK); anti-TOMM20 (sc-17764) and anti-HA (sc-7392) were purchased from Santa Cruz Biotechnology (Dallas, TX, USA); anti-LC3 (NB100-2220), anti-HIF-1α (NB100-479), and anti-HIF-2α (NB100-122) antibodies were purchased from NOVUS Biologicals (Centennial, CO, USA); and anti-ACTA1 (MAB1501) was purchased from Sigma. For protein detection, the membranes were incubated with HRP-conjugated secondary antibodies (7076S and 7074S; Cell Signaling Technology, Danvers, MA, USA).

### 4.7. Immunoprecipitation

For the immunoprecipitation assay, cells were lysed for 1 h at 4 °C by using the RIPA buffer (50 mM Tris–HCl, pH 7.5, 150 mM sodium chloride, 0.5% sodium deoxycholate, 1% Triton X-100, 0.1% SDS, and 2 mM EDTA) (IBS-BR002, Intron Biotechnology, Seongnam, Republic of Korea) containing protease inhibitors (P3100, GenDEPOT). The supernatant was immunoprecipitated with anti-ABCD3 (sc-514728; Santa Cruz Biotechnology), which was added to the lysate for 24 h at 4 °C. Then, protein A/G PLUS-agarose (sc-2003; Santa Cruz Biotechnology) was added, and the samples were incubated for 2–4 h at 4 °C. All the cell lysates were prepared by using the 2× Laemmli sample buffer (1610737; Bio-Rad). Finally, all the samples were analyzed via Western blotting.

### 4.8. Quantification and Statistical Analysis

For fluorescent image analyses, the free software ImageJ Fiji 64-bit (NIH) was used to calculate the number of peroxisomes and perform a double-fluorescence correlation coefficient analysis. Data were obtained from at least three independent experiments and are presented as the mean ± standard error of the mean. Statistical evaluation of the results was performed by using a one-way analysis of variance. *p*-values of <0.001 were considered to indicate statistical significance, whereas p-values of >0.05 were considered to indicate statistical nonsignificance.

## Data Availability

The data presented in this study are available in article.
